# Comparative occurrence and antibiogram of extended-spectrum β-lactamase-producing *Escherichia coli* among post-weaned calves and lactating cows from smallholder dairy farms in a parallel animal husbandry area

**DOI:** 10.14202/vetworld.2021.1311-1318

**Published:** 2021-05-26

**Authors:** Chya Vannakovida, Kannika Na Lampang, Phongsakorn Chuammitri, Veerasak Punyapornwithaya, Khwanchai Kreausukon, Raktham Mektrirat

**Affiliations:** 1 Department of Veterinary Bioscience and Veterinary Public Health, Faculty of Veterinary Medicine, Chiang Mai University, Chiang Mai 50100, Thailand; 2 Department of Food Animal Clinic, Faculty of Veterinary Medicine, Chiang Mai University, Chiang Mai 50100, Thailand; 3 Epidemiology Research Group of Infectious Disease, Chiang Mai University, Chiang Mai 50200, Thailand

**Keywords:** antibiogram, antimicrobial resistance, cattle, *Escherichia coli*, extended-spectrum β-lactamase, smallholder dairy farm

## Abstract

**Background and Aim:**

Inappropriate overuse of antimicrobials might be associated with the spreading of antimicrobial-resistant bacteria in animal-based food products. Extended-spectrum β-lactamase (ESBL)-producing *Escherichia coli* have been recognized as an emerging global problem in a One Health approach. This study aimed to assess the occurrence and antimicrobial-susceptible profiles of ESBL-producing *E. coli* among post-weaned calves and lactating cows in a parallel animal husbandry area.

**Materials and Methods:**

Seventy-two pool fecal samples were collected from 36 smallholder dairy farms registered in Ban Hong Dairy Cooperatives, Lamphun Province, Thailand. Pre-enriched fecal samples were cultured in MacConkey agar supplemented with cefotaxime. The potential *E. coli* isolates were identified by not only biochemical tests but also polymerase chain reaction assay of the *16S rRNA* gene. ESBL production was confirmed by the combination disk test. Antimicrobial susceptibility testing was performed by the Kirby–Bauer disk diffusion method.

**Results:**

The occurrence of ESBL-producing *E. coli* at the farm level was 80.56%. The different phenotypic antibiogram of ESBL-producing *E. coli* was observed among post-weaned calf and lactating cow specimens. The most frequent resistance patterns of ESBL-producing isolates from both groups were amoxicillin-ceftiofur-cephalexin-cephalothin-cloxacillin-streptomycin-oxytetracycline-sulfamethoxazole/trimethoprim. For the median zone diameter, enrofloxacin-resistant isolates with narrow zone diameter values from lactating cow specimens were particularly more than post-weaned calf specimens **(**p**<**0**.**05**).**

**Conclusion:**

These findings revealed the dynamic changes in ESBL-producing *E. coli* from calves and lactating cows in Lamphun Province, posing the inevitability to prevent bacterial transmission and optimize antimicrobial therapy in dairy farming.

## Introduction

Antimicrobial resistance (AMR) has occurred worldwide, with consequent economic impact and public health concerns [1,2]. AMR is a comprehensive problem related to humans, animals, and the environment. Because the global situation is fast progressing into a post-antibiotic era, urgent strategic plans are essential for surveillance and prevention to avert the AMR crisis. At present, the AMR mechanism of bacterial enzyme-mediated resistance to antimicrobial agents is well documented. In particular, β-lactamase enzymes (EC 3.5.2.6) produced by Gram-negative bacteria that hydrolyze the covalent bond of the β-lactam ring structure are the primary resistance mechanism to β-lactam antimicrobials [3]. Extended-spectrum β-lactamase (ESBL)-producing *Escherichia coli* are the commensal and pathogenic bacteria in the critical priority group classified according to the greatest threat to human health and the urgent need for new antimicrobials by the World Health Organization (WHO) [4]. ESBL enzymes can confer bacterial resistance to penicillin, first- to third-generation cephalosporin, and monobactam that are inhibited by β-lactamase inhibitors, such as clavulanic acid, sulbactam, and tazobactam [5]. In 2017, the Centers for Disease Control and Prevention have reported that ESBL-producing *Enterobacteriaceae* threats in the United States had approximately 197,000 morbidity cases and 9100 deaths [6]. Moreover, many reports have shown that ESBL-producing *E. coli* infections lead to increased morbidity and mortality rates, treatment failure, requiring more complex treatments, prolonged hospital stays, and limited therapeutic options [4-6]. Nevertheless, ESBL-producing *E. coli* occur in healthcare settings and are present in the communities. During the past decade, these problems have recently raised significant concerns in the human food chain. Several reports have published ESBL-producing *E. coli* detected in farm animals, including pigs, poultry, and cattle [7-9].

Cattle meat and dairy products are the primary food protein sources unquestionably required in the human diet [10,11]. Unfortunately, animal fecal mass is the major source of gut microflora as a reservoir and disseminates ESBL-producing *E. coli* [12]. The environmental contamination of ESBL-producing *E. coli* creates a consensual concern in the scientific community and the One Health approach [13,14]. Of additional importance, *E. coli* producing CTX-M-2 β-lactamase in cattle has been previously described in Japan [15]. From the first description to the present, ESBL-producing *Enterobacteriaceae* have recently been reported in cattle production in 40 countries [16-19]. Despite this significant problem, information on the occurrence and dissemination of ESBL among calves and cows is lacking, whereas ongoing surveillance has previously provided some information on antimicrobial use [20] in bulls, cows, and calves [21]. In addition, the excessive and inappropriate antimicrobial use in both calves and cows has been considered one of the main contributors to the selection of ESBL-producing *E. coli*.

The Thailand situation report on ESBL-producing *E. coli* in dairy cattle is seldom published. Moreover, the presence of ESBL-producing *E. coli* in the development status of cattle farming is still very limited. Consequently, this study aimed to assess the occurrence of ESBL-producing *E. coli* from pooled fecal samples from healthy calves and cows in smallholder dairy farms in Lamphun Province, Thailand. No data are available on the antibiograms of multidrug-resistant (MDR) bacteria or ESBL-producing *E. coli* in healthy cattle from smallholder dairy farms in a parallel animal husbandry area. Therefore, the antimicrobial susceptibility profile of ESBL-producing isolates was also evaluated.

## Materials and Methods

### Ethical approval

The research study was ethically approved by the Animal Care and Use Committee, Faculty of Veterinary Medicine, Chiang Mai University (approval number: S3/2562). Holstein Friesian cows were housed in free-stall barn dairy farms and milked twice a day. Cows with good udder health were required for this study. The Institute Biosafety Committee, Chiang Mai University, also granted permission to test the pathogens (approval number: CMU A-0762019).

### Study period and location

The study was conducted from April to May 2020 in 36 smallholder dairy farms registered in Ban Hong Dairy Cooperatives, Lamphun Province, Thailand.

### Sample population and collection

The mean herd sizes were 30 lactating cows (max=80, min=6) and 25 post-weaned calves (max=86, min=6). On each farm, the fecal samples were taken through rectal palpation of all dairy cattle by the veterinarian. In addition, 10 fecal samples from lactating dairy cows were included in one pooled fecal sample and five fecal samples from calves were collected for one pooled fecal sample. Both pooled fecal samples from healthy calves and lactating cows were assembled on the same farm. All samples were kept at 5°C and transported to the laboratory within 6 h. On the same day of sample collection, the dairy farmers answered a questionnaire on general information, such as demographic data, antimicrobial use, and calf feeding.

### Microbiological identification of ESBL-producing E. coli

Pooled fecal samples (5 g) were cultured in 45 mL pre-enrichment Luria-Bertani (LB) [22] broth (HiMedia, India). After incubation at 37°C for 24 h, a loop full of the pre-enrichment cultures was streaked onto MacConkey agar (HiMedia, India) supplemented with 1 mg/L cefotaxime. The suspected pink colonies with precipitated bile on MacConkey agar containing cefotaxime were identified to be *E. coli* using standard IMIViC biochemical tests, including indole production test, methyl red test, Voges–Proskauer test, and citrate utilization test. These tests also included triple sugar iron test and urease test. The *16S rRNA* gene of strains of *E. coli* was also determined using previously published primers **[**23**].** Then, *E. coli* isolates were measured as ESBL production by the combination disk test (CDT) using cefotaxime (30 μg), cefotaxime/clavulanic acid (30/10 μg), ceftazidime (30 μg), and ceftazidime/clavulanic acid (30/10 μg) [24]. The phenotypic ESBL isolates were confirmed when the inhibition zone of cephalosporins combined with clavulanic acid was ≥5 mm compared to cephalosporins alone. In addition, *E. coli* (ATCC 25922) and *Klebsiella pneumoniae* (ATCC 700603) were used as the quality negative control strain and positive ESBL control type strain, respectively.

### Antimicrobial susceptibility testing

Antimicrobial susceptibility testing was performed by the Kirby–Bauer disk diffusion method according to CLSI [24]. ESBL-producing *E. coli* isolates were streaked on 5% sheep blood agar plates and incubated at 37°C for 24 h. Bacterial culture was adjusted to a concentration of 1.5 × 10^8^ colony-forming units/mL in Mueller-Hinton broth using a McFarland densitometer (Grant Instruments, Cambridgeshire, UK) and swabbed on BBL™ Mueller-Hinton agar plates (Becton Dickinson & Co., Sparks, MD, USA). All ESBL-producing *E. coli* isolates were subjected to the antimicrobial susceptibility test by the Kirby–Bauer disk diffusion method with 14 antimicrobial agents (Table-1). After 24 h incubation at 30°C, the size of the bacterial growth inhibition zones was interpreted as sensitive (S), intermediately resistant (I), or resistant (R) according to the antimicrobial breakpoints for *Enterobacteriaceae* by the CLSI guidelines [24,25].

**Table-1:** Antimicrobials disks and their concentrations and breakpoints used for the Kirby–Bauer disk diffusion method (CLSI M100, 2014; CLSI VET01, 2018).

Antimicrobial agent	Conc. (µg)	Breakpoint (mm)	

		S	R
Amoxicillin	10	≥17	≤13
Amoxicillin/clavulanic acid	30/10	≥18	≤13
Ceftiofur	30	≥21	≤17
Cephalexin	30	≥18	≤14
Cephalothin	30	≥18	≤14
Cloxacillin	5	≥13	≤10
Chloramphenicol	50	≥18	≤12
Enrofloxacin	5	≥23	≤16
Gentamicin	10	≥15	≤12
Kanamycin	30	≥18	≤13
Streptomycin	10	≥15	≤11
Oxytetracycline	30	≥15	≤11
Imipenem	5	≥23	≤19
Sulfamethoxazole/trimethoprim	23.75/1.25	≥16	≤10

### Data management and statistical analysis

The geographical distribution of selected smallholder dairy farms was demonstrated by mapping using Quantum Information System version 2**.**18**.**28**.** Descriptive statistics were used to describe data, including frequency, percentage, proportion to express the general characteristics, and basic information on the occurrence of ESBL-producing *E. coli* and its antibiogram, to compare the main outcome differences between the calf and cow groups. The antimicrobial susceptibility of ESBL-producing *E. coli* was interpreted qualitatively as resistant, intermediate, or susceptible. The isolates with resistant phenotype to three or more antimicrobial classes were identified as MDR. The AMR patterns of ESBL-producing *E. coli* isolates were summarized in terms of frequencies. The distribution of inhibition zone diameters of antimicrobials against ESBL-producing *E. coli* was plotted, and the trendlines of cumulative curves were performed in sixth-degree polynomial approximation. By the point of interception, the median (ZD_50_) and 90^th^ percentile (ZD_90_) were also calculated by polynomial regression equations. The Mann–Whitney test was also used for comparison between the calf and cow groups with a non-normal distribution. The differences between variables were considered statistically significant when the bicaudal probability of their occurrence due to chance (error type I) was lower than 5% (p<0.05). Statistical analysis was performed with R statistical software (RStudio, Boston, MA, USA).

## Results

**Figure-1:**
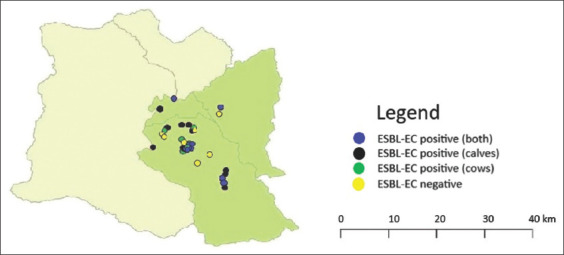
Geographic distribution of ESBL-producing *Escherichia coli* isolates in 36 selected dairy farms located in Ban Hong dairy cooperatives, Lamphun Province, Thailand, at the status level. Mapping was performed in Quantum Geographic Information System version 2.18.28. ESBL-EC=extended-spectrum β-lactamase-producing *E. coli*.

All pooled fecal samples from the calf and cow groups were pre-enriched in LB broth. Sixty-eight of the 72 samples (94.44%) were culture positive for the screening of cefotaxime-resistant *Enterobacteriaceae*. Subsequently, 40 ESBL-producing *E. coli* isolates were confirmed by CDT. At the sample level of the pooled fecal samples, ESBL-producing *E. coli* were found in 55.56% (40/72) of the total samples. At least one positive sample in either the calf group or the cow group for ESBL-producing *E. coli* was counted as the occurrence of values within the farm level. In addition, the occurrence frequency of ESBL-producing *E. coli* at the farm level was 80.56% (29/36 farms). Positive ESBL-producing *E. coli* in both calf and cow fecal samples were found on 11 farms (30.56%). Nine farms only detected positive ESBL-producing *E. coli* in calf fecal samples (25.00%), whereas nine other farms detected positive ESBL-producing *E. coli* in cow fecal samples (25.00%; Table-2). The geographical distribution of ESBL-producing *E. coli* isolates of dairy farms at the farm level and at the status level is shown in Figure-1.

**Table-2:** The occurrence and 95% confidence interval (95% CI) of ESBL-producing *E. coli* at the farm level and the sample level from fecal samples of healthy calves and cows in smallholder dairy farms, Lamphun, Thailand, during April-May 2020.

Numbers of positive sample	Farm level (n=36)	95% CI	Sample level (n=72)	95% CI
Either calf or cow	80.56% (29/36)	63.43-91.20	55.56% (40/72)	43.41-67.10
Both calf and cow	30.56% (11/36)	16.92-48.27	-	-
Only calf	25.00% (9/36)	12.73-42.54	-	-
Only cow	25.00% (9/36)	12.73-42.54	-	-
Neither calf nor cow	19.44% (7/36)	8.80-36.57	44.44% (32/72)	32.90-56.59

**Figure-2:**
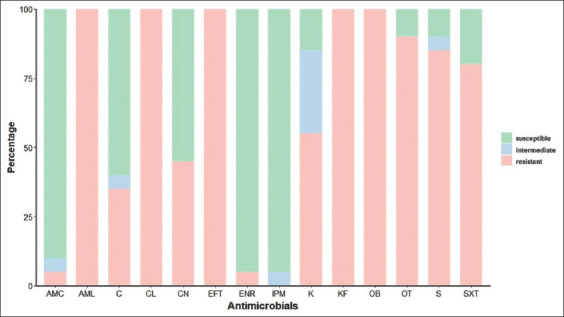
Antibiogram profiles of extended-spectrum β-lactamase-producing *Escherichia coli* collected from pooled fecal samples on healthy calves, in Lamphun (n = 20 isolates distributed in different farms). Abbreviations: AMC=amoxicillin/clavulanic acid, AML=amoxicillin, C=chloramphenicol, CL=cephalexin, CN=gentamicin, EFT=ceftiofur, ENR=enrofloxacin, IPM=imipenem, K=kanamycin, KF=cephalothin, OB=cloxacillin, OT=oxytetracycline, S=streptomycin, SXT=sulfamethoxazole/trimethoprim.

In ESBL-producing *E. coli* from calf fecal samples results, 20 isolates were completely resistant (100%) to amoxicillin, ceftiofur, cephalexin, cephalothin, and cloxacillin. A high level (50-99%) of drug resistance to oxytetracycline, streptomycin, sulfamethoxazole/trimethoprim, and kanamycin was observed. In contrast, a low level (1-49%) of drug resistance to gentamicin, chloramphenicol, enrofloxacin, and amoxicillin/clavulanic acid was also found. No ESBL-producing *E. coli* isolates were resistant to imipenem (Figure-2).

**Figure-3:**
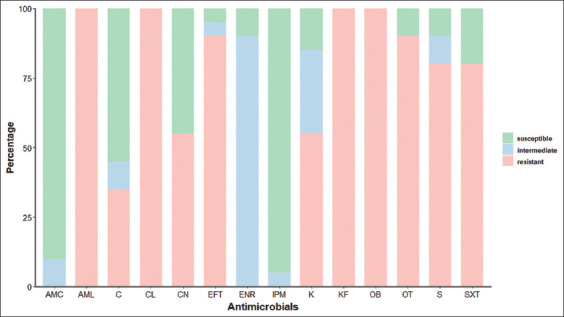
Antibiogram profiles of extended-spectrum β-lactamase-producing *Escherichia coli* collected from pooled fecal samples on healthy cows, in Lamphun (n = 20 isolates distributed in different farms). Abbreviations: AMC=amoxicillin/clavulanic acid, AML=amoxicillin, C=chloramphenicol, CL=cephalexin, CN=gentamicin, EFT=ceftiofur, ENR=enrofloxacin, IPM=imipenem, K=kanamycin, KF=cephalothin, OB=cloxacillin, OT=oxytetracycline, S=streptomycin, SXT=sulfamethoxazole/trimethoprim.

In ESBL-producing *E. coli* from cow fecal samples results, 20 ESBL-producing *E. coli* isolates were completely resistant (100%) to amoxicillin, cephalexin, cephalothin, and cloxacillin. A high level (50-99%) of drug resistance to oxytetracycline, ceftiofur, streptomycin, sulfamethoxazole/trimethoprim, gentamicin, and kanamycin was observed. In contrast, a low level (1-49%) of drug resistance to chloramphenicol was also found. No ESBL-producing E. coli isolates were resistant to amoxicillin/clavulanic acid, enrofloxacin, and imipenem (Figure-3).

ESBL-producing *E. coli* isolates were tested against nine groups of antimicrobials, and resistance to at least three groups indicated that the isolates were MDR. All isolates collected from healthy calves and cows were resistant to at least three classes. In all, 30.0% of ESBL-producing *E. coli* isolates were resistant to eight and nine antimicrobial agents, and 10% showed resistance to seven antimicrobial agents. The most frequent resistance patterns of ESBL-producing *E. coli* isolated from calf groups (four isolates) and cow groups (three isolates) (Table-3).

**Table-3:** Resistance patterns of ESBL-producing *E. coli* in healthy calves and cows.

No. of agents	Antimicrobial resistance pattern	Calves	Cows
12	AML-EFT-CL-KF-OB-C-ENR-CN-K-S-OT-SXT	1	0
11	AML-EFT-CL-KF-OB-C-CN-K-S-OT-SXT	3	0
10	AML-EFT-CL-KF-OB-CN-K-S-OT-SXT	1	2
	AML-EFT-CL-KF-OB-C-K-S-OT-SXT	1	0
	AML-EFT-CL-KF-OB-C-CN-S-OT-SXT	0	2
	AML-EFT-CL-KF-OB-C-CN-K-OT-SXT	0	2
9	AML-EFT-CL-KF-OB-C-K-OT-SXT	1	0
	AML-EFT-CL-KF-OB-CN-S-OT-SXT	3	1
	AML-EFT-CL-KF-OB-C-CN-K-S	0	1
	AML-EFT-CL-KF-OB-C-S-OT-SXT	0	2
	AML-EFT-CL-KF-OB-CN-K-S-OT	0	2
	AML-EFT-CL-KF-OB-K-S-OT-SXT	2	0
8	AML-AMC-EFT-CL-KF-OB-K-OT	1	0
	AML-EFT-CL-KF-OB-C-S-OT	1	0
	AML-EFT-CL-KF-OB-CN-OT-SXT	0	1
	AML-CL-KF-OB-K-S-OT-SXT	0	2
	AML-EFT-CL-KF-OB-S-OT-SXT	4	3
7	AML-EFT-CL-KF-OB-CN-K	1	0
	AML-CL-KF-OB-S-OT-SXT	0	1
	AML-EFT-CL-KF-OB-K-S	0	1
6	AML-EFT-CL-KF-OB-S	1	0

AML=Amoxicillin, AMC=Amoxicillin/clavulanic acid, EFT=Ceftiofur, CL=Cephalexin, KF=Cephalothin, OB=Cloxacillin, C=Chloramphenicol, ENR=Enrofloxacin, CN=Gentamicin, S=Streptomycin, OT=Oxytetracycline, IPM=Imipenem, SXT=Sulfamethoxazole/trimethoprim, *ESBL=*Extended-spectrum β-lactamase

**Figure-4:**
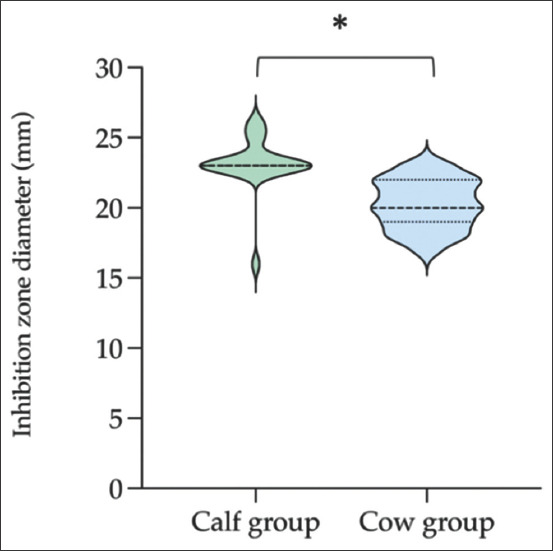
Distribution of inhibition zone diameters for enrofloxacin against extended-spectrum β-lactamase-producing *Escherichia coli* isolates among healthy calves and cows. Violin plots extend from the fist to the third quartiles, the thick bar in the center represents the median.

By the point of interception, the distribution of inhibition zone diameters of ESBL-producing *E. coli* isolates was described in the ZD_50_ and ZD_90_ values calculated by polynomial regression equations**.** In both calf and cow groups, the ZD_50_ and ZD_90_ values for amoxicillin, cephalexin, cephalothin, and cloxacillin were 0 mm. The resistance was eventually seen to almost all antimicrobial inhibition diameters that were tested (Table-4). However, most antimicrobial ZD_90_ values in the cow group were broader than the cow group, except for kanamycin, streptomycin, oxytetracycline, and imipenem. For ZD_50_, most antimicrobial diameters in the calf group were broader than the cow group, except for chloramphenicol, gentamicin, oxytetracycline, and sulfamethoxazole/trimethoprim. Interestingly, various inhibition diameters for enrofloxacin against ESBL-producing *E. coli* isolates were observed among the calf group (16-26 mm) and the cow group (17-23 mm). Moreover, the median zone diameter for enrofloxacin against ESBL-producing *E. coli* isolates was 23 (*interquartile range* [IQR], 23-23) for the calf group and 20 (IQR, 19-22) for the cow group (*P* < 0.05, Mann–Whitney test) (Figure-4).

**Table-4:** The ZD_50_ and ZD_90_ values of this study among different 10 antimicrobials of ESBL-producing E. coli isolated from healthy calves and cows.

Antimicrobial	Conc. (µg)	Calf group		Cow group	
	
		^1^ZD_50_	^2^ZD_90_	ZD_50_	ZD_90_
Amoxicillin/clavulanic acid	30/10	10.47	10.00	13.44	13.30
Ceftiofur	30	9.25	6.62	9.24	6.69
Chloramphenicol	50	13.34	9.76	14.86	10.94
Enrofloxacin	5	23.49	15.76	16.98	15.97
Gentamicin	10	8.22	8.04	10.29	8.77
Kanamycin	30	15.32	15.16	15.29	12.76
Streptomycin	10	17.89	17.80	13.71	13.55
Oxytetracycline	30	12.16	7.06	19.84	6.04
Imipenem	5	8.70	8.70	7.83	7.83
Sulfamethoxazole/trimethoprim	23.75/ 1.25	26.11	23.86	27.76	27.04

1 ZD_50_, mean zone diameter (in mm) over which 50% of the isolates were inhibited.

2 ZD_90_, mean zone diameter (in mm) over which 90% of the isolates were inhibited. *ESBL=*Extended-spectrum β-lactamase

## Discussion

This research was designed to determine the presence of ESBL-producing *E. coli* on dairy cattle from smallholder farms in Lamphun Province and elucidate the antibiograms from pooled fecal samples from healthy calves and cows in a parallel animal husbandry area. The ESBL-producing *E. coli* status was defined among calf and cow specimens at the herd level; therefore, individual animal aspects were not assessed. In a previous study, ESBL-producing *E. coli* strains could be isolated more than twice using enrichment procedures [19]. Consequently, in this study, the pooled fecal samples were pre-enriched in LB broth and cultured in MacConkey agar supplemented with cefotaxime to screen cefotaxime-resistant *Enterobacteriaceae*. Before ESBL phenotypic confirmation using the CDT, potential E. coli isolates were identified by not only biochemical tests but also polymerase chain reaction (PCR) assay. Based on the approaches described in Materials and Methods, the molecular characterization of antimicrobial-resistant *E. coli* isolated from domestic and food-producing animals was widely tested for the presence of *16S rRNA* gene using PCR methods [26,27].

In smallholder farms in Lamphun Province, a high occurrence of ESBL-producing *E. coli* at the farm level was observed (80.56%). These findings agree with previous reports on the percentage of ESBL-producing *E. coli* (86.7%)-positive samples in Bavarian dairy and beef cattle farms in Germany [19]. Subsequently, the presence of ESBL-producing *E. coli* strains in dairy cattle was evaluated in distinct countries, and the occurrence frequency of resistance varied in individual regions. In contrast, a low occurrence of ESBL-producing *E. coli* from cattle farming was reported in the Netherlands (59.6%) [28], China (43.6%) [29], Great Britain (37.5%) [30], Germany (18.0%) [7], and Finland (2.0%) [31]. Interestingly, this study demonstrated that one-half occurrence of ESBL-producing *E. coli* was found in either calf or cow group (55.56%). This result was in contrast to the previous epidemiological studies in Germany [19], Switzerland [18], and Great Britain [30] that the prevalence of ESBL-producing *E. coli* in calves was higher than in cows. In this study, the geographical distribution of ESBL-producing *E. coli* in 36 dairy farms located in Ban Hong Dairy Cooperatives was conducted. The global positioning system of this subregion coordinates is 18°19′35.1″N, 98°46′36.6″E. The average temperature range is 20.7*-*33.0°C (69.26-91.4°F), and the average humidity is 72.16*%*. Interestingly, this area is consistent with recently published studies on the occurrence of ESBL-producing *E. coli* in healthy pigs (76.7-98.06%) [9,32], healthy poultry (40.0%) [32], and healthy humans (77.30-96.52%) [9,32].

The Kirby–Bauer test for the antimicrobial susceptibility to ESBL producer isolates was interpreted qualitatively as resistant, intermediate, or susceptible. This study demonstrated that ESBL-producing *E. coli* isolates from both calf and cow specimens were predominantly inhibited by imipenem and amoxicillin/clavulanic acid. However, stewardship efforts of preserving the imipenem are a prerequisite [33]. All ESBL-producing *E. coli* isolates showed a high rate of resistance against β-lactam antimicrobials, oxytetracycline, sulfamethoxazole/trimethoprim, and streptomycin. Moreover, the results demonstrated that all isolates of ESBL-producing *E. coli* were resistant to at least three classes from healthy calves and healthy cows. The previous studies have shown that all ESBL-producing E. coli isolates from healthy or sick dairy cattle (e.g. diarrhea and mastitis) were commonly present as MDR [34-36]. Besides, resistance to β-lactam antimicrobials and resistance to tetracycline, macrolide, sulfonamide, and diaminopyrimidine were the most common resistance pattern among ESBL-producing E. coli isolates. ESBL resistance genes selected by non-β-lactams were previously documented [8]. Paterson and Bonomo also revealed that using various antimicrobial classes, including sulfamethoxazole/trimethoprim, quinolones, and aminoglycosides, are associated with subsequent infections due to ESBL-producing bacteria [5]. These resistant antimicrobials in this study have been routinely used to treat and prevent disease in dairy cattle worldwide and also in Thailand. The alimentary tract of ruminants has a great quantity of bacteria as the normal commensal flora [20]. For *Enterobacteriaceae* generally considered as commensal alimentary inhabitants, AMR genes for ESBLs could potentially be horizontally transferred among acquired resistance bacteria [8,37,38]. Therefore, there is a higher probability for non-pathogenic commensal *E. coli* to become a reservoir of AMR in the food chain.

Interestingly, the interpretive results of enrofloxacin susceptibility testing of ESBL-producing *E. coli* from calf specimens with susceptible phenotype, in contrast to isolates from cow specimens with intermediate phenotype, were observed. Moreover, similar data were obtained with regard to the sensitivity of the tested isolates to enrofloxacin. The ZD_50_ value from the calf group was entirely in the sensitivity zone, and the range of inhibition zone diameters was markedly broader than the cow group. Increasing enrofloxacin resistance may also pose a significant threat to animal health and food safety. In food-producing animals, mutations within the chromosomal target site of gyrA and parC in *E. coli* were described [39,40]. Moreover, the most common plasmid-borne resistance mechanism is also the mutation of the plasmid-mediated quinolone resistance gene of oqxAB [41]. Fluoroquinolones are recommended for use in non-lactating dairy calve for the treatment or control of diarrhea and respiratory disease. A previous study demonstrated the increased resistance of *E. coli* after using enrofloxacin in calves [42]. Treatment of pre-weaned calves at high risk for bovine respiratory disease with enrofloxacin resulted in a significant increase in the shedding of ciprofloxacin-resistant *E. coli* in feces for up to 7 days after medication [43]. These study findings may have differed from the current study due to the use of different pre-weaning and post-weaning groups. Additional differences could be particularly attributed to the bacterial strains of non-ESBL and ESBL producers.

In 2001, the WHO published that the inappropriate use of antimicrobials (dose, duration, and indication) might increase the overall selective pressure of AMR pathogens [44]. During the past decade, several studies in many countries, including Thailand, have generally reported that commensal *E. coli* in food-producing animals are becoming more resistant to antimicrobials (7-9). Therefore, it is important to continuously monitor the antimicrobial susceptibility profiles of *E. coli* isolated from food-producing animals in practices that result in resistant antimicrobials important in human and animal medicine.

## Conclusion

The results highlighted the first study on ESBL-producing *E. coli* in herd status in dairy farms in Lamphun Province, Thailand. This research indicated that the different phenotypic antibiogram of ESBL-producing *E. coli* was observed among post-weaned calf and lactating cow specimens. Interestingly, enrofloxacin-resistant isolates with narrow zone diameter values from lactating cow specimens were particularly more than post-weaned calf specimens**.** Further studies are necessary to deepen the epidemiological knowledge on AMR in the dairy food chain. Continuous monitoring of antimicrobial susceptibility profiles should be performed to improve antimicrobial stewardship in animal farming and ensure final product safety.

## Authors’ Contributions

KNL, KK, and RM: Designed the study concept and the research experiments. CV: Performed the sample collection and laboratory testing. RM, KNL, PC, and VP: Conducted the formal analysis and the data curation. CV and RM: Prepared the original draft of the manuscript. RM: Contributed the scientific advice, review, and editing. All authors read and approved the final manuscript.

## References

[1] Reygaert W.C (2018). An overview of the antimicrobial resistance mechanisms of bacteria. AIMS Microbiol.

[2] World Health Organization (2014). Antimicrobial Resistance:Global Report on Surveillance.

[3] Rawat D, Nair D (2010). Extended-spectrum β-lactamases in gram-negative bacteria. J. Glob. Infect. Dis.

[4] Tacconelli E, Magrini N, Kahlmeter G, Singh N (2017). Global priority list of antibiotic-resistant bacteria to guide research, discovery, and development of new antibiotics. World Health Organ.

[5] Paterson D.L, Bonomo R.A (2005). Extended-spectrum beta-lactamases:A clinical update. Clin. Microbiol. Rev.

[6] Centers for Disease Control Prevention (2020). Antibiotic Resistance Threats in the United States 2019.

[7] Dahms C, Hübner N.O, Kossow A, Mellmann A, Dittmann K, Kramer A (2015). Occurrence of ESBL-producing *Escherichia coli*in livestock and farm workers in Mecklenburg-Western Pomerania, Germany. PLoS One.

[8] Poirel L, Madec J.Y, Lupo A, Schink A.K, Kieffer N, Nordmann P, Schwarz S (2018). Antimicrobial resistance in *Escherichia coli*. Microbiol. Spectr.

[9] Seenama C, Thamlikitkul V, Ratthawongjirakul P (2019). Multilocus sequence typing and bla (ESBL) characterization of extended-spectrum beta-lactamase-producing *Escherichia coli* isolated from healthy humans and swine in Northern Thailand. Infect. Drug Resist.

[10] Bruinsma J (2003). World Agriculture:Towards 2015/2030:An FAO Perspective:Earthscan.

[11] Ritchie H, Roser M (2017). Meat and Dairy Production. Our World in Data.

[12] Smith K, Williams A (2016). Production and management of cattle manure in the UK and implications for land application practice. Soil Use Manage.

[13] Booton R.D, Meeyai A, Alhusein N, Buller H, Feil E, Lambert H, Mongkolsuk S, Pitchforth E, Reyher K.K, Sakcamduang W, Satayavivad J, Singer A.C, Sringernyuang L, Thamlikitkul V, Vass L, Avison M.B, Turner K.M.E, OH-DART Study Group (2021). One health drivers of antibacterial resistance:Quantifying the relative impacts of human, animal and environmental use and transmission. One Health.

[14] Horton R.A, Randall L.P, Snary E.L, Cockrem H, Lotz S, Wearing H, Duncan D, Rabie A, McLaren I, Watson E, La Ragione R.M, Coldham N.G (2011). Fecal carriage and shedding density of CTX-M extended-spectrum β-lactamase-producing *Escherichia coli* in cattle, chickens, and pigs:Implications for environmental contamination and food production. Appl. Environ. Microbiol.

[15] Shiraki Y, Shibata N, Doi Y, Arakawa Y (2004). *Escherichia coli* producing CTX-M-2 beta-lactamase in cattle, Japan. Emerg. Infect. Dis.

[16] Ali T, Ur Rahman S, Zhang L, Shahid M, Zhang S, Liu G, Gao J, Han B (2016). ESBL-Producing *Escherichia coli* from cows suffering mastitis in China contain clinical class 1 integrons with CTX-M linked to ISCR1. Front. Microbiol.

[17] Dantas P.J, Ferreira H.M.N (2020). Extended-spectrum beta-lactamase (ESBL)-producing *Enterobacteriaceae* in cattle production a threat around the world. Heliyon.

[18] Geser N, Stephan R, Hächler H (2012). Occurrence and characteristics of extended-spectrum β-lactamase (ESBL) producing *Enterobacteriaceae* in food-producing animals, minced meat and raw milk. BMC Vet. Res.

[19] Schmid A, Hörmansdorfer S, Messelhäusser U, Käsbohrer A, Sauter-Louis C, Mansfeld R (2013). Prevalence of extended-spectrum β-lactamase-producing *Escherichia coli* on Bavarian dairy and beef cattle farms. Appl. Environ. Microbiol.

[20] Henderson G, Cox F, Ganesh S, Jonker A, Young W, Janssen P.H, Global Rumen Census Collaborators (2015). Rumen microbial community composition varies with diet and host, but a core microbiome is found across a wide geographical range. Sci. Rep.

[21] Waldner C.L, Parker S, Gow S, Wilson D.J, Campbell J.R (2019). Antimicrobial usage in western Canadian cow-calf herds. Can. Vet. J.

[22] Aidara-Kane A, Angulo F.J, Conly J.M, Minato Y, Silbergeld E.K, McEwen S.A, Collignon P.J, WHO Guideline Development Group (2018). World Health Organization (WHO) guidelines on use of medically important antimicrobials in food-producing animals. Antimicrob. Resist. Infect. Control.

[23] Tsen H, Lin C, Chi W (1998). Development and use of *16S rRNA* gene targeted PCR primers for the identification of *Escherichia coli* cells in water. J. Appl. Microbiol.

[24] Clinical and Laboratory Standards Institute (2014). Performance Standards for Antimicrobial Susceptibility Testing.

[25] Clinical and Laboratory Standards Institute (2018). Performance Standards for Antimicrobial Disk and Dilution Susceptibility Tests for Bacteria Isolated from Animals.

[26] Chung Y.S, Park Y.K, Park Y.H, Park K.T (2017). Probable secondary transmission of antimicrobial-resistant *Escherichia coli* between people living with and without pets. J. Vet. Med. Sci.

[27] Zhang Y, Zhou J, Dong Z, Li G, Wang J, Li Y, Wan D, Yang H, Yin Y (2019). Effect of dietary copper on intestinal microbiota and antimicrobial resistance profiles of *Escherichia coli* in weaned piglets. Front. Microbiol.

[28] Heuvelink A.E, Gonggrijp M.A, Buter R.G.J, ter Bogt-Kappert C.C, van Schaik G, Velthuis A.G.J, Lam T.J.G (2019). Prevalence of extended-spectrum and AmpC β-lactamase-producing *Escherichia coli* in Dutch dairy herds. Vet Microbiol.

[29] Zheng B, Feng C, Xu H, Yu X, Guo L, Jiang X, Song X (2018). Detection and characterization of ESBL-producing *Escherichia coli* expressing mcr-1 from dairy cows in China. J. Antimicrob. Chemother.

[30] Velasova M, Smith R.P, Lemma F, Horton R.A, Duggett N.A, Evans J, Tongue S.C, Anjum M.F, Randall L.P (2015). Detection of extended-spectrum β-lactam, AmpC and carbapenem resistance in *Enterobacteriaceae* in beef cattle in Great Britain in 2015. J. Appl. Microbiol.

[31] Päivärinta M, Pohjola L, Fredriksson-Ahomaa M, Heikinheimo A (2016). Low occurrence of extended-spectrum β-lactamase-producing *Escherichia coli* in Finnish food-producing animals. Zoonoses Public Health.

[32] Boonyasiri A, Tangkoskul T, Seenama C, Saiyarin J, Tiengrim S, Thamlikitkul V (2014). Prevalence of antibiotic-resistant bacteria in healthy adults, foods, food animals, and the environment in selected areas in Thailand. Pathog. Glob. Health.

[33] Vickers R.J, Bassetti M, Clancy C.J, Garey K.W, Greenberg D.E, Nguyen M.H, Roblin D, Tillotson G.S, Wilcox M.H (2019). Combating resistance while maintaining innovation:The future of antimicrobial stewardship. Future Microbiol.

[34] Ali T, Rahman S.U, Zhang L, Shahid M, Han D, Gao J, Zhang S, Ruegg P.L, Saddique U, Han B (2017). Characteristics and genetic diversity of multi-drug resistant extended-spectrum beta-lactamase (ESBL)-producing *Escherichia coli* isolated from bovine mastitis. Oncotarget.

[35] Ewers C, Stamm I, Stolle I, Guenther S, Kopp P.A, Fruth A, Wieler L.H, Scheufen S, Bauerfeind R, Bethe A, Prenger-Berninghoff E (2014). Detection of Shiga toxin and extended-spectrum β-lactamase-producing *Escherichia coli* O145:NM and Ont:NM from calves with diarrhoea. J. Antimicrob. Chemother.

[36] Tark D.S, Moon D.C, Kang H.Y, Kim S.R, Nam H.M, Lee H.S, Jung S.C, Lim S.K (2017). Antimicrobial susceptibility and characterization of extended-spectrum β-lactamases in *Escherichia coli* isolated from bovine mastitic milk in South Korea from 2012 to 2015. J. Dairy Sci.

[37] Mesa R.J, Blanc V, Blanch A.R, Cortés P, González J.J, Lavilla S, Miró E, Muniesa M, Saco M, Tórtola M.T, Mirelis B, Coll P, Llagostera M, Prats G, Navarro F (2006). Extended-spectrum β-lactamase-producing *Enterobacteriaceae* in different environments (humans, food, animal farms and sewage). J. Antimicrob. Chemother.

[38] Pereira R.V, Altier C, Siler J.D, Mann S, Jordan D, Warnick L.D (2020). Longitudinal effects of enrofloxacin or tulathromycin use in preweaned calves at high risk of bovine respiratory disease on the shedding of antimicrobial-resistant fecal *Escherichia coli*. J. Dairy Sci.

[39] Liu B.T, Liao X.P, Yang S.S, Wang X.M, Li L.L, Sun J, Yang Y.R, Fang L.X, Li L, Zhao D.H, Liu Y.H (2012). Detection of mutations in the gyrA and parC genes in *Escherichia coli* isolates carrying plasmid-mediated quinolone resistance genes from diseased food-producing animals. J. Med. Microbiol.

[40] Redgrave L.S, Sutton S.B, Webber M.A, Piddock L.J (2014). Fluoroquinolone resistance:Mechanisms, impact on bacteria, and role in evolutionary success. Trends Microbiol.

[41] Liu B.T, Yang Q.E, Li L, Sun J, Liao X.P, Fang L.X, Yang S.S, Deng H, Liu Y.H (2013). Dissemination and characterization of plasmids carrying oqxAB-blaCTX-M Genes in *Escherichia coli* isolates from food-producing animals. PLoS One.

[42] Pereira R, Siler J, Ng J, Davis M, Warnick L (2014). Effect of preweaned dairy calf housing system on antimicrobial resistance in commensal *Escherichia coli*. J. Dairy Sci.

[43] World Health Organization (2002). WHO Global Strategy for Containment of Antimicrobial Resistance.

